# Prognostic value of serum free light chain abnormalities in diffuse large B-cell lymphoma

**DOI:** 10.3389/fonc.2025.1694343

**Published:** 2025-12-08

**Authors:** Zhuxia Jia, Jin Li, Jie Liu, Wenmin Han, Wei Xu, Xuzhang Lu

**Affiliations:** 1 Department of Hematology, The Second People’s Hospital of Changzhou, The Third Affiliated Hospital of Nanjing Medical University, Changzhou, China; 2 The Changzhou Medical Center of Nanjing Medical University, Changzhou, China; 3 Department of Hematology, The First Affiliated Hospital of Nanjing Medical University, Nanjing, China

**Keywords:** diffuse large B-cell lymphoma, international prognostic index, overall survival, prognostic biomarker, serum free light chain

## Abstract

**Introduction:**

This study aimed to evaluate the prognostic significance of serum free light chain (FLC) abnormalities in patients diagnosed with diffuse large B-cell lymphoma (DLBCL), with an emphasis on associations with clinical characteristics and survival outcomes.

**Methods:**

This retrospective study included 259 patients newly diagnosed with DLBCL. Serum FLC levels in patients diagnosed with DLBCL were measured prior to the initiation of chemotherapy.

**Results:**

Comparing to normal FLC, abnormal serum FLC levels were significantly associated with several clinical features including age > 60 years (*p* = 0.01), advanced diseased stage (stage III/IV, *p* < 0.001), involvement of multiple extra nodal sites (*p* = 0.019), elevated lactate dehydrogenase (LDH) (*p* = 0.005), Eastern Cooperative Oncology Group performance status (ECOG PS) scores of >1 *(p* = 0.02), high-intermediate to high International Prognostic Index (IPI) scores (*p* = 0.005), bone marrow infiltration (*p* = 0.001), presence of B symptoms, and a higher prevalence of non-germinal center B-cell (non-GCB) subtype. While monoclonal FLC expression was associated with advanced disease stages (stage III/IV, *p* < 0.001), high-intermediate to high IPI scores (*p* < 0.001), bone marrow infiltration (*p* < 0.001), elevated LDH (*p* < 0.001), and categorization within high-risk prognostic groups. Distinct clinical characteristics were also observed between patients with normal and abnormal serum FLC levels based on molecular classification. Among 259 patients, patients with abnormal or monoclonal FLC profiles had significantly reduced three-year overall survival (OS) and progression-free survival (PFS) (*p* = 0.016 and 0.032 and *p* = 0.008 and 0.024, respectively) compared to normal FLC profiles. Among patients with non-GCB DLBCL, monoclonal FLC was associated with a significantly reduced three-year OS compared to normal FLC levels (*p* = 0.008). Results of multivariate Cox regression analysis for FLC as an independent prognostic factor were not statistically significant.

**Conclusions:**

Abnormal serum FLC levels were associated with adverse clinical features and inferior survival outcomes in DLBCL, particularly monoclonal FLC in the non-GCB subtype. These findings support the utility of serum FLC assessment as a simple, accessible biomarker for risk stratification in DLBCL, though it was not confirmed as an independent risk factor in multivariate analysis.

## Introduction

Diffuse large B-cell lymphoma (DLBCL) represents the most prevalent subtype of non-Hodgkin lymphoma. Following first-line immunotherapy with rituximab, cyclophosphamide, doxorubicin (hydroxydaunorubicin), vincristine (Oncovin), and prednisone (R-CHOP), the five-year progression-free survival (PFS) and overall survival (OS) rates are 60% and 65%, respectively (1). The International Prognostic Index (IPI) is a clinical tool for evaluating prognosis in individuals diagnosed with invasive diseases (2). However, significant heterogeneity persists within the subgroups underscoring the need for accessible and reliable biomarkers to support prognosis prediction in patients undergoing rituximab-based chemotherapy.

Serum free light chain (sFLCs) are produced during normal immunoglobulin synthesis by B-cells and plasma cells. Due to imbalances in heavy and light chain synthesis, surplus kappa (κ) or lambda (λ) light chains may be secreted into the blood stream. A normal FLC profile includes quantitative levels of κFLC, λFLC, and the κ:λ ratio. Abnormal FLC profiles are categorized as polyclonal—characterized by elevated levels of one or both light chains with a normal κ:λ ratio—or monoclonal, which is defined by an abnormal κ:λ ratio. Polyclonal elevations are observed in conditions associated with B-cell hyperactivity, immune stimulation, or renal impairment (3, 4). Monoclonal FLC expression has established prognostic and diagnostic utility in disorders such as multiple myeloma and amyloidosis (5, 6). Abnormal FLC profiles have also been documented in several lymphoma subtypes, including Hodgkin lymphoma and mantle cell lymphoma (7, 8). In the context of primary central nervous system lymphoma (PCNSL), the κ:λ ratio in cerebrospinal fluid may serve as a novel biomarker for early diagnosis and monitoring of chemotherapy efficacy (9). Abnormal FLC expression has also been associated with adverse prognosis in individuals diagnosed with DLBCL (10). Although previous studies have confirmed that serum free light chain (sFLC) abnormalities are associated with adverse prognosis in diffuse large B-cell lymphoma (DLBCL) (10), the association analysis between DLBCL molecular subtypes (especially GCB/non-GCB subtypes based on the Hans criteria) and sFLC abnormalities remains fragmented, lacking systematic exploration of the correlation among “molecular subtype-FLC abnormal pattern-prognosis”.

Based on this, this study, by strictly excluding special DLBCL subtypes and plasma cell diseases, systematically analyzed the association between “polyclonal/monoclonal sFLC abnormalities” and clinical characteristics of DLBCL (such as Ann Arbor stage, IPI score, and bone marrow infiltration) in a large retrospective cohort (n=259) for the first time. Furthermore, it explored the prognostic value of sFLC abnormalities in GCB/non-GCB subtypes through stratified analysis, aiming to fill the research gap in “FLC abnormal subtype-molecular subtype-prognosis” and provide a more accurate biomarker basis for risk stratification of DLBCL.

## Materials and methods

### Study participants

This retrospective study included 259 patients newly diagnosed with DLBCL, who were hospitalized at Changzhou Second People’s Hospital and Jiangsu Provincial People’s Hospital between January 2017 and June 2022. The cohort comprised of 131 males and 128 females, with a median age of 61 years (range: 20–89 years). Histopathological confirmation of DLBCL was required for inclusion. All patients were detected for myc rearrangement, BCL2 rearrangement, and BCL6 rearrangement using FISH method, and high-grade B-cell lymphoma HGBL was excluded. Patients diagnosed with primary mediastinal (thymic) large B-cell lymphoma, primary central nervous system DLBCL, post-transplant lymphoproliferative disorders, primary cutaneous DLBCL, Monoclonal Gammopathy of Undetermined Significance (MGUS) or other plasma cell dyscrasias were excluded from the study. Additional exclusion criteria included the presence of severe infections, cardiovascular diseases, or rheumatic immune diseases.

Immunohistochemical (IHC) staining was performed on formalin-fixed, paraffin-embedded (FFPE) tissue sections of DLBCL samples to determine the cell-of-origin (COO) subtype. The Hans criteria were applied for classification into germinal center B-cell-like (GCB) or non-germinal center B-cell-like (non-GCB) subtypes. Briefly, IHC markers including CD10, BCL6, and MUM1 were detected using specific primary antibodies and a standard streptavidin-biotin-peroxidase complex (SABC) method. GCB subtype was defined as positive staining for CD10 (regardless of BCL6 and MUM1 expression) OR positive for BCL6 and negative for MUM1 (if CD10 was negative). Non-GCB subtype was defined as negative for both CD10 and BCL6, OR positive for MUM1 (regardless of CD10 expression if BCL6 was negative). Staining results were independently evaluated by two experienced hematopathologists. Discrepancies were resolved through joint review and consensus to ensure diagnostic accuracy.

All participants underwent comprehensive baseline assessments, including enhanced computed tomography (CT) or positron emission tomography-computed tomography (PET-CT), bone marrow examination, LDH testing, and other related investigations. IPI was used for prognostic stratification, with 1 point for each of the following criteria age > 60 years, stage III/IV disease, elevated LDH level, ECOG PS scores ≥ 2, and involvement of more than one extra nodal site. Based on IPI scores, participants were categorized into four risk groups: low risk (score 0–1), low-intermediate risk (score 2), high-intermediate risk (score 3), and high-risk (score 4–5). Written informed consent was obtained from all participants.

### Free light chain detection

sFLCs were measured prior to each chemotherapy cycle. Blood samples were collected in coagulation-promoting tubes and centrifuged at 3,500 revolutions per minute for 15 minutes. A minimum of 450 µl of serum was then transferred into sample cups. Samples were organized according to identification numbers and placed on racks for automatic analysis. Reagents, including FLC detection reagents, sample diluents, and rinse solutions, were preloaded into the reagent compartment of the instrument prior to testing.

The detection principle is based on the immunoturbidimetric method: the specific anti-κ and anti-λ monoclonal antibodies in the reagent specifically bind to κ and λ FLC in the serum sample, forming insoluble immune complexes. The formation of these complexes causes an increase in the turbidity of the reaction system, and the degree of turbidity increase has a linear positive correlation with the concentration of κ and λ FLC in the serum within a certain range. The instrument automatically detects the turbidity change of the reaction system at a specific wavelength (630 nm), and calculates the exact concentration of κ FLC and λ FLC in the serum sample (unit: mg/l) by comparing with the standard curve established by the standard substance with known FLC concentration.

Elevated serum κ or λ FLC concentrations were defined as κ >19.4 mg/l or λ >26.3 mg/l. The normal κ:λ FLC ratio ranges from 0.26–1.65. This was elevated to 0.37–3.1 in patients diagnosed with renal failure. An increased concentration of either κ or λ FLC was considered indicative of FLC elevation.

Polyclonal FLC abnormalities were defined by elevated κ and/or λ FLC levels with a normal κ:λ ratio. Monoclonal FLC abnormalities were defined by an abnormal κ:λ ratio. Both polyclonal and monoclonal patterns were categorized as abnormal FLC profiles.

### Statistical analyses

OS was defined as the time from diagnosis until death from any cause. PFS was defined as the interval from the date of diagnosis to either disease progression or death from any cause. Categorical variables (e.g., Ann Arbor stage, IPI risk group, presence of B symptoms, and FLC status) were compared using the Chi-square test, when the expected frequency of any cell in the contingency table was less than 5, the Fisher’s exact test was used instead to ensure accurate statistical inference. Survival outcomes were evaluated using the Kaplan–Meier method and compared using the log-rank test. The prognostic significance of clinical and biochemical variables on OS and PFS were assessed using univariate and multivariate Cox regression analysis. A *p*-value of < 0.05 was considered statistically significant. All statistical analyses were conducted using SPSS for Windows, version 26.0 (IBM Corp., Armonk, NY, USA).

## Results

### Baseline characteristics

Among the 259 patients newly diagnosed with DLBCL, 95 (36.7%) were classified as having the germinal center B-cell-like (GCB) subtype and 164 (63.3%) had the non-GCB subtype. The cohort consisted of 144 males (55.6%) and 115 females (44.4%), with a median age of 61 (range: 20–89 years). A total of 131 patients (50.6%) were aged over 60 years. A total of 156 patients (60.2%) presented with stage III/IV disease, 72 patients (27.8%) had more than one external node involvement, 112 patients (43.2%) had elevated LDH levels, and 34 patients (13.1%) had bone marrow infiltration. According to the IPI scoring system, 91 patients (35.1%) were classified in the high-intermediate and high-risk groups, while 168 patients (64.9%) were classified into the low and low-intermediate risk groups. Elevated creatinine levels were observed in 6 patients (2.3%) (**Table 1**).

**Table 1:** Patient characteristics based on all patients and normal or abnormal FLC.

Clinical characteristics	All patients (*n* = 259)	Normal FLC (*n* = 166)	Abnormal FLC (*n* = 93)	*S* value	*P* value
Age >60 years, *n*(%)	131(50.6)	74(44.6)	57(61.3)	6.660	0.010
Male sex, *n* (%)	144(55.6)	92(55.4)	52(55.9)	0.006	0.939
Stage III/IV, *n* (%)	156(60.2)	81(48.8)	75(80.6)	25.243	<0.001
B symptom, *n* (%)	76(29.3)	40(24.1)	36(38.7)	6.139	0.013
ECOG>1 *n* (%)	38(14.7)	18(10.8)	20(21.5)	5.412	0.020
IPI HI/H, *n* (%)	91(35.1)	42(25.3)	49(52.7)	19.617	<0.001
HAS(N), *n* (%)	164(63.3)	96(57.8)	68(73.1)	5.997	0.014
Extranodal >1, n (%)	72(27.8)	38(22.9)	34(36.6)	5.548	0.019
Bone marrow invasion *n*(%)	34(13.1)	13(7.8)	21(22.6)	11.370	0.001
Ki67 > 90% *n* (%)	70(27.0)	46(27.7)	24(25.8)	0.110	0.741
LDH>UNL, *n* (%)	112(43.2)	61(36.7)	51(54.8)	7.949	0.005

FLC, free light chain; ECOG, Eastern Cooperative Oncology Group performance status; IPI, International Prognostic Index; HAS, Hans Algorithm; LDH, Lactate Dehydrogenase; UNL, Upper Normal Limit.

### Clinical characteristics based on abnormal and normal FLC profiles

Among the 259 patients diagnosed with DLBCL, the median serum concentrations of κFLC and λFLC were 13.5 mg/l (range: 5.5–712.5) and 20.4 mg/l (range: 5.47–392.5), respectively. A total of 166 patients exhibited normal FLC profiles, while 93 patients (35.9%) demonstrated abnormal FLC values. Among those, 69 patients (26.6%) demonstrated elevated κ and/or λ FLC levels with a normal κ:λ ratio (polyclonal FLC), and 24 patients (9.3%) demonstrated an abnormal κ:λ FLC ratio (monoclonal FLC). Of note, 58 patients (22.4%) had elevated κFLC concentrations, while 69 patients (26.6%) had elevated λFLC levels reported in the dataset.

The presence of abnormal FLCs was associated with several clinical factors, including age > 60 years (*p* = 0.01), advanced disease stage (Ann Arbor stage III/IV, *p* < 0.001), involvement of more than one extra nodal site (*p* = 0.019), elevated LDH levels (*p* = 0.005), ECOG PS >1 (*p* = 0.02), high-intermediate or high IPI scores (*p* = 0.005), bone marrow infiltration (*p* = 0.001), presence of B symptoms, and a higher frequency of the non-GCB subtype. (**Table 1**).

Comparative analysis among patients with monoclonal, polyclonal, and normal FLC profiles indicated statistically significant differences in several clinical parameters, including age, Ann Arbor stage, presence of B symptoms, IPI score, non-GCB subtype distribution, bone marrow infiltration, and LDH elevation (**Table 2**). When comparing polyclonal FLC to normal FLC profiles, only Ann Arbor stage (*p* < 0.001) and high-intermediate/high IPI scores (*p* < 0.005) showed statistically significant differences. In contrast, monoclonal FLC profiles were significantly associated with advanced disease stage (*p* < 0.001), high-intermediate/high IPI scores (*p* < 0.001), bone marrow infiltration (*p* < 0.001), elevated LDH levels (*p* < 0.001), and classification into high risk prognostic groups.

**Table 2:** Patient characteristics based on normal, monoclonal, and polyclonal FLC.

Clinical characteristics	All patients(n= 259)	Normal FLC (n= 166)	Polyclonal FLC (n= 69)	Monoclonal FLC (n=24)	S value	P value
Age >60 years, n (%)	131(50.6)	74(44.6)	42(60.7)	15(62.5)	6.679	0.035
Male sex, n (%)	144(55.6)	92(55.4)	38(55.1)	14(58.3)	0.083	0.960
Stage III/IV, n (%)	156(60.2)	81(48.8)	52(75.4)	23(95.8)	28.359	<0.001
B symptom, n (%)	76(29.3)	40(24.1)	25(36.2)	11(45.8)	6.931	0.031
ECOG>1 n (%)	38(14.7)	18(10.8)	14(20.3)	6(25)	5.442	0.066
IPI HI/H, n (%)	91(35.1)	42(25.3)	32(46.4)	17(70.8)	27.452	<0.001
HAS(N), n (%)	164(63.3)	96(57.8)	50(73.5)	18(75)	6.047	0.049
Extranodal >1, n (%)	72(27.8)	38(22.9)	26(37.7)	8(33.3)	5.715	0.057
Bone marrow invasion n(%)	34(13.1)	13(7.8)	10(14.5)	11(45.8)	26.707	<0.001
Ki 67 > 90% n (%)	70(27.0)	46(27.7)	19(27.5)	5(20.8)	0.615	0.735
LDH>UNL, n (%)	112(43.2)	61(36.7)	32(46.4)	19(79.2)	15.750	<0.001

FLC, free light chain; ECOG, Eastern Cooperative Oncology Group performance status; IPI, International Prognostic Index; HAS, Hans Algorithm; LDH, Lactate Dehydrogenase; UNL, Upper Normal Limit.

### Clinical characteristics according to molecular subtypes

Among patients with the non-GCB DLBCL, abnormal FLC status was significantly associated with an advanced disease stage (Ann Arbor stage III/IV, *p* = 0.004), high-intermediate/high IPI scores (IPI 3–5, *p* = 0.04), and bone marrow infiltration (*p* = 0.041), when compared with those with normal FLC profiles. Further subgroup comparisons among monoclonal, polyclonal, and normal FLC profiles within the non-GCB cohort revealed significant differences in Ann Arbor stage (*p* = 0.002), IPI score (*p* = 0.014), bone marrow infiltration (*p* = 0.005), and LDH levels (*p* = 0.002). When comparing polyclonal to normal FLC profiles specifically, only high-intermediate to high IPI scores differed significantly (*p* < 0.001). Compared with the normal FLC group, monoclonal FLC was associated with advanced disease stage (stage III/IV, *p* = 0.003), high-intermediate/high IPI scores (*p* = 0.004), bone marrow infiltration (*p* < 0.001), and elevated LDH levels (*p* = 0.002).

In the GCB subtype, abnormal FLC status was significantly associated with age > 60 years (*p* = 0.01), advanced disease stage (Ann Arbor stage III/IV, *p* < 0.001), involvement of more than one extra nodal site (*p* = 0.048), ECOG PS status >1 (*p* = 0.004), high-intermediate to high IPI scores (*p* < 0.001), bone marrow infiltration (*p* = 0.013), and the presence of B symptoms (*p* = 0.032).

Given that only 25 patients in the GCB group exhibited abnormal FLC values, no other subgroup analyses were performed.

### OS and PFS

Of the 259 patients diagnosed with DLBCL, 68 patients were lost to follow-up. The remaining 191 patients, all of whom received R-CHOP as first-line treatment, were included in the survival analysis. The mean follow-up duration for OS and PFS was 48.30 months (range: 45.93–50.67) and 46.95 months (range: 44.36–49.54), respectively. A total of 34 (17.8%) patients died during the follow-up period. The three-year OS and PFS rates were 82% and 80%, respectively.

**Figure 1:**
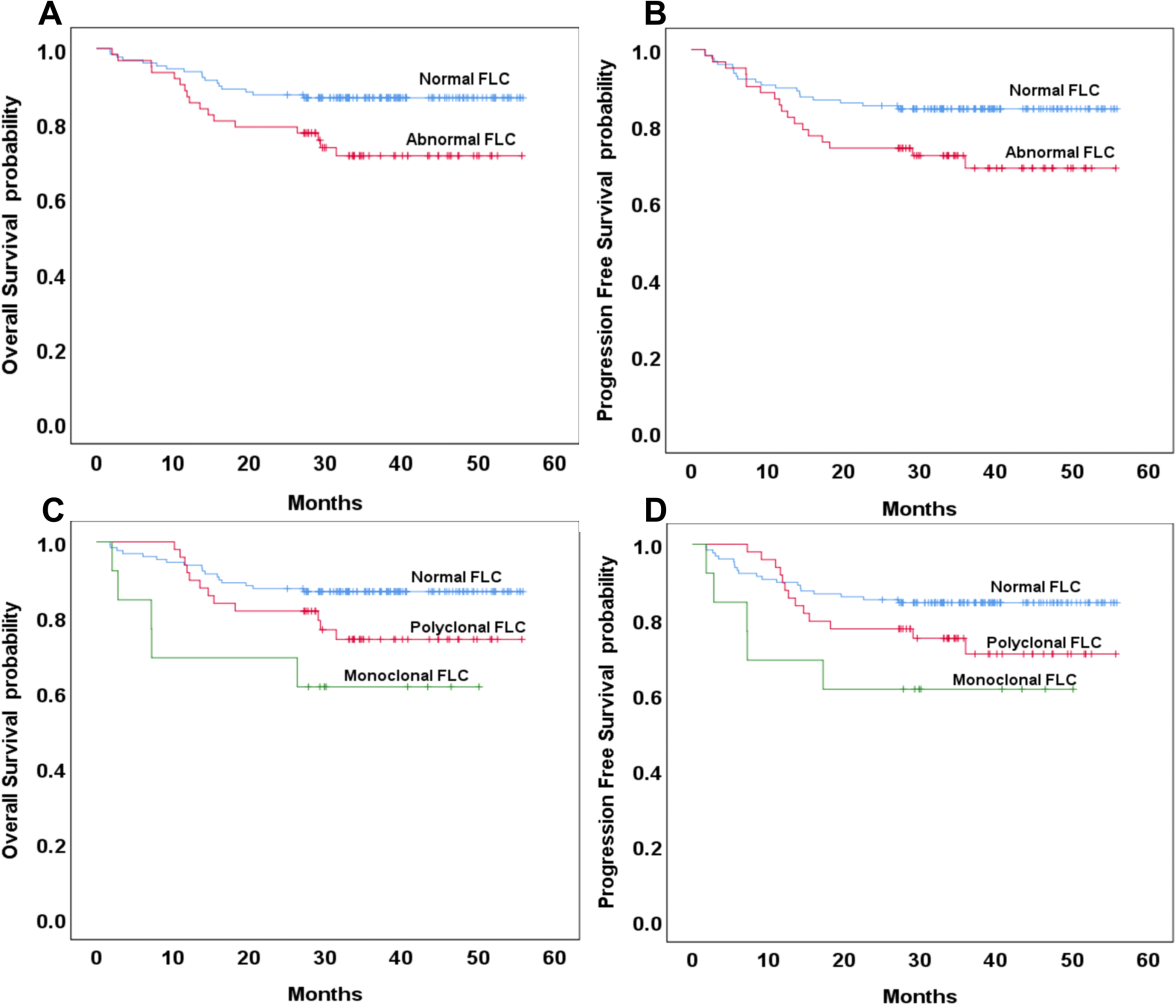
Overall and progression-free survival based on normal or abnormal serum free light chain (FLC) **(A, B)** and the presence of monoclonal or polyclonal FLC **(C, D)**.

Patients with abnormal FLC profiles exhibited significantly lower three-year OS and PFS rates compared to those with normal FLC profiles. Specifically, the three-year OS was 71% in the abnormal FLC group versus 87% in the normal group (p = 0.016; hazard ratio [HR]: 2.24; 95% confidence interval [CI]: 47.52–52.74 vs. 39.51–49.00), and the three-year PFS was 69% versus 84% (p = 0.032; HR: 1.98; 95% CI: 45.88–51.71 vs. 37.82–47.95) (**Figure 1A, B**). No significant differences in three-year OS or PFS was observed between patients with the normal and polyclonal FLC profiles. The three-year OS was 87% in both groups (p = 0.076; HR: 1.92; 95% CI: 47.52–52.74 vs. 41.17–50.85), and the three-year PFS was 84% in the normal group versus 71% in the polyclonal group (p = 0.115; HR: 1.73; 95% CI: 45.88–51.71 vs. 39.32–49.80) (**Figure 1C, D**).

In contrast, patients with monoclonal FLC profiles had significantly lower three-year OS and PFS compared to those with normal FLC profiles. The three-year OS was 62% in the monoclonal group versus 87% in the normal group (p = 0.008; HR: 2.24; 95% CI: 23.13–45.64 vs. 47.52–52.74), and the three-year PFS was also 62% versus 84%, respectively (p = 0.024; HR: 3.05; 95% CI: 22.18–45.16 vs. 45.88–51.71). There were no significant differences in OS or PFS between patients with polyclonal and monoclonal FLC profiles (*p* = 0.174 and 0.261, respectively) (**Figure 1C, D**).

### OS and PFS based on molecular classification

**Figure 2:**
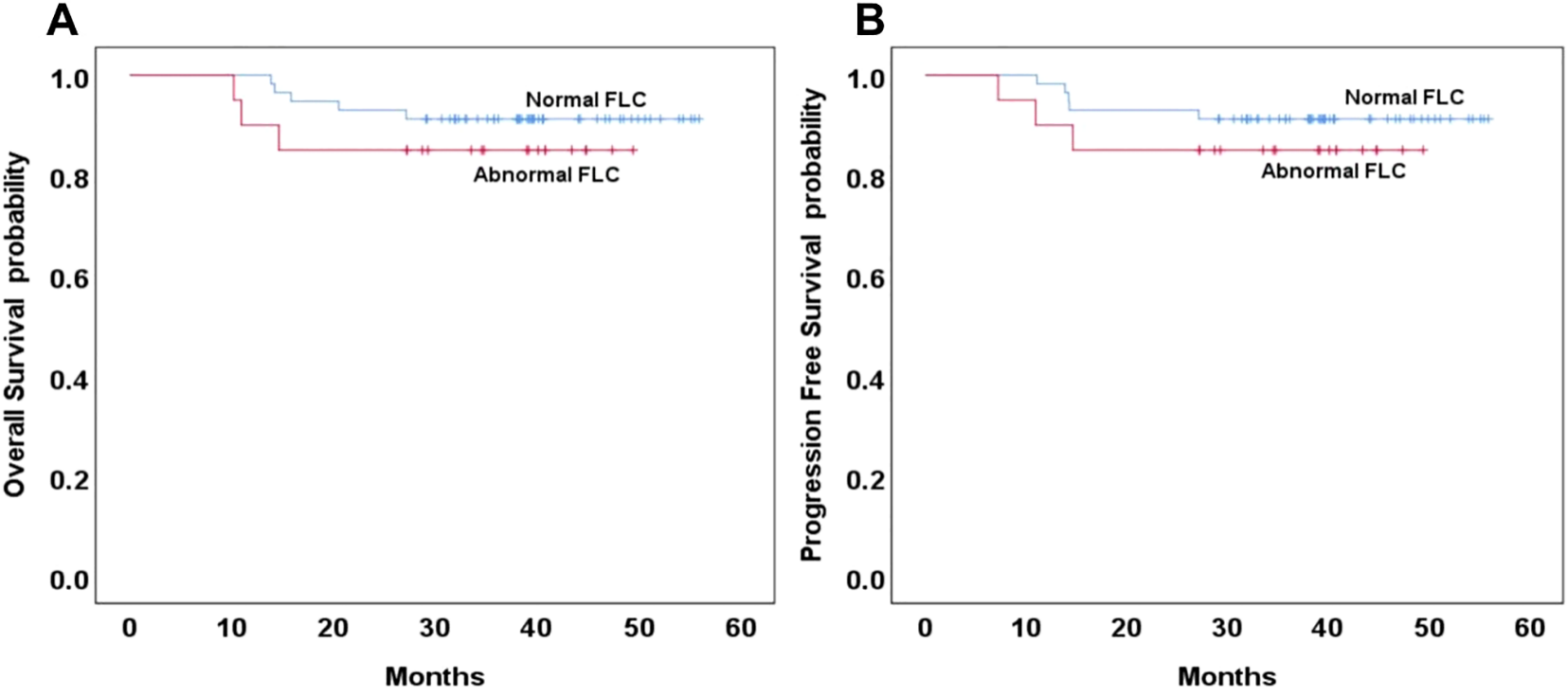
Overall and progression-free survival based on normal or abnormal in the GCB type of DLBCL **(A, B)**.

**Figure 3:**
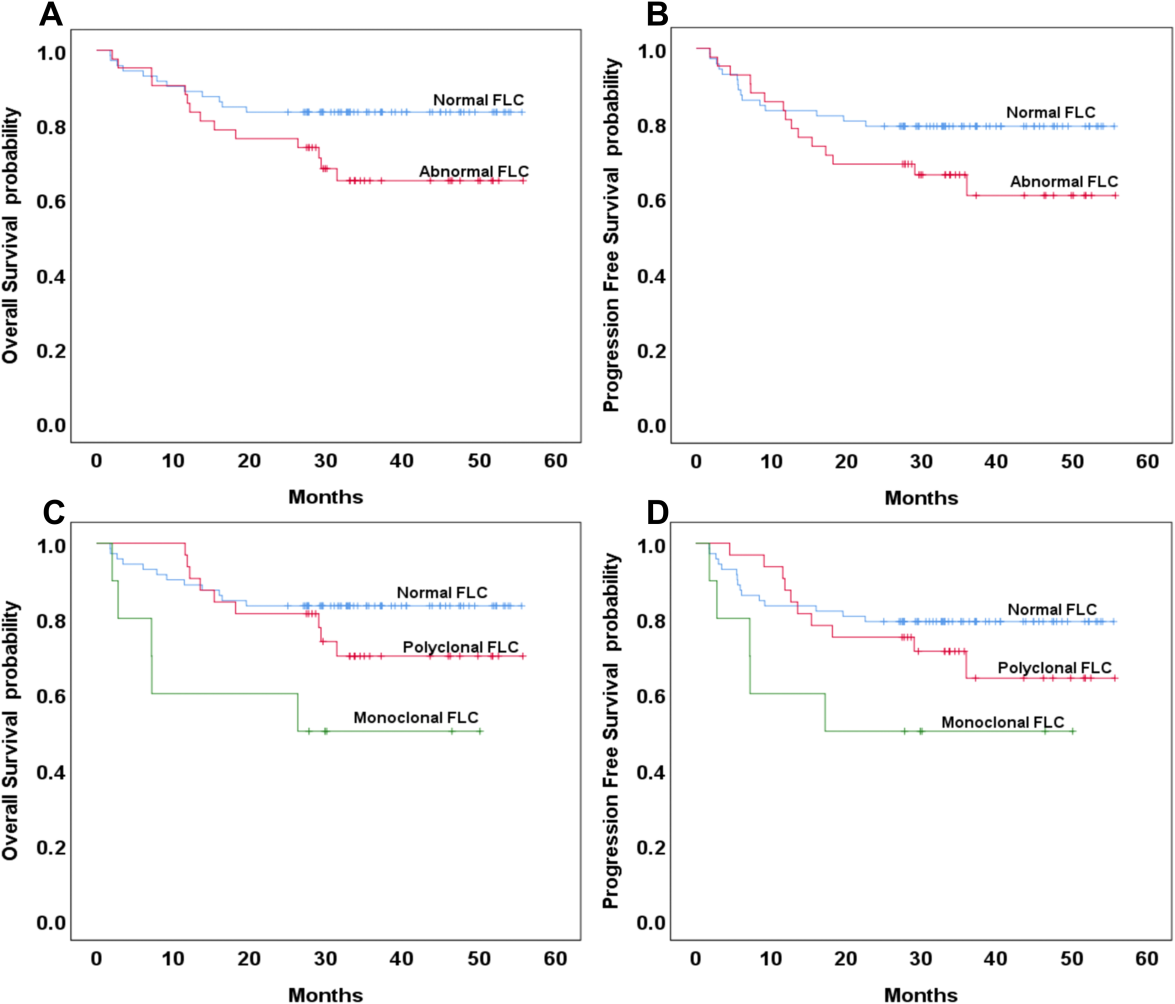
Overall survival and progression-free survival based on normal or abnormal **(A, B)** and the presence of monoclonal or polyclonal FLC **(C, D)** in the non-GCB type of DLBCL.

Among the 191 patients included in the survival analysis, 77 (40.3%) were classified as having the GCB subtype of DLBCL. Of these, 20 patients (35.3%) exhibited abnormal FLC profiles. Of the 20 patients with abnormal FLC, only 3 (3.9%, 3/77) had monoclonal FLC abnormalities. No statistically significant differences were observed in three-year OS and PFS between patients with normal and abnormal FLC profiles in the GCB subgroup (*p* = 0.391 and 0.407, respectively; **Figure 2A, B**). Among the 114 patients (59.7%) with the non-GCB subtype of DLBCL, 42 (36.8%) exhibited abnormal FLC profiles, comprising 32 patients with polyclonal FLC and 10 patients (8.8%) with monoclonal FLC. The distribution of abnormal and monoclonal FLC profiles did not differ significantly by molecular subtype classification (*p* = 0.116 and 0.189, respectively). In the non-GCB subgroup, the three-year OS was 83% in the normal FLC group compared to 65% in the abnormal FLC group, with the difference approaching but not reaching statistical significance *(p* = 0.054). Similarly, the three-year PFS was 79% and 61%, respectively (*p* = 0.113), (**Figure 3A, B**). When comparing normal and polyclonal FLC groups, the three-year OS was 83% versus 70%, respectively (*p* = 0.248; hazard ratio [HR]: 1.65; 95% confidence interval [CI]: 43.83–51.90 vs. 38.96–51.03). The three-year PFS also did not differ significantly between these groups (79% vs. 64%, *p* = 0.076; HR: 1.46; 95% CI: 41.12–50.18 vs. 35.57–49.32) (**Figure 3C, D**).

However, patients with monoclonal FLC had significantly reduced three-year OS compared to those with normal FLC profiles (83% vs. 50%, *p* = 0.008; HR: 4.104; 95% CI: 43.83–51.90 vs. 16.36–42.95). Although the three-year PFS was also lower in the monoclonal group (79% vs. 50%), the difference did not reach statistical significance (*p* = 0.076; HR: 3.064; 95% CI: 41.12–50.18 vs. 15.21–42.22) (**Figure 3C, D**).

No statistically significant differences were observed between polyclonal and monoclonal FLC groups in terms of three-year OS (*p* = 0.072) or PFS (*p* = 0.076) (**Figure 3C, D**).

### Clinical characteristics of monoclonal FLC

Among the 259 patients diagnosed with DLBCL, monoclonal free light chain (FLC) abnormalities were identified in 24 patients (9.27%). Of these, 23 patients demonstrated elevated κ and/or λ FLC concentrations. The majority of patients with monoclonal FLC abnormalities were over 60 years of age (15/24; 62.5%) and nearly all presented with stage III/IV disease (23/24; 95.8%). A total of 17 patients (70.8%) had high-intermediate/high IPI scores. Follow-up FLC measurements were conducted after the second cycle of chemotherapy in 18 patients with monoclonal FLC. Of these, 11 patients (61.1%) demonstrated normalization of the κ:λ ratio. Among the 13 patients with monoclonal FLC abnormalities who were included in the survival analysis, 5 (38.4%) died during the follow-up period.

## Discussion

Abnormal secretion of FLCs is frequently observed in untreated lymphomas, with prevalence varying with lymphoma subtype (11). Previous studies on individuals with DLBCL have reported elevated serum FLC levels in 32% to 54.9% of cases, and monoclonal FLC abnormalities in 14% to 22.9% of cases (12, 13). In the present cohort of 259 patients with DLBCL, 35.9% and 9.3% of patients exhibited abnormal FLC levels and monoclonal FLC abnormalities, respectively.

Elevated polyclonal FLC levels may result from immune disruption or stimulation or renal dysfunction (3, 4). In this cohort, all six patients with abnormal renal function demonstrated elevated FLC concentrations, though statistical analysis was not conducted due to the small sample size. Abnormal FLC profiles were associated with older age and poor clinical outcomes. Both polyclonal and monoclonal FLC elevations were more frequently observed in patients with advanced-stage disease and high intermediate to high IPI scores. Additionally, patients with monoclonal FLC abnormalities exhibited a higher prevalence of elevated LDH levels and bone marrow infiltration, indicating a potential association between monoclonal FLC expression and increased tumor burden.

A study by Witzig et al. indicated that individuals with DLBCL and abnormal serum FLC profiles had lower PFS and OS rates compared with those with normal FLC profiles, which is consistent with the findings of this study (14). The source of FLC production may vary by malignancy and is influenced by the tumor microenvironment. In multiple myeloma, monoclonal plasma cells are responsible for FLC secretion, while in Hodgkin lymphoma, polyclonal FLCs are produced by benign tumor microenvironment cells simulated by the tumor (8). In DLBCL, both polyclonal and monoclonal FLC elevations have been associated with adverse clinical outcomes, regardless of IPI score. However, individuals with monoclonal FLC abnormalities tend to have the worst prognosis (14).

This study found that in DLBCL patients, those with abnormal FLC profiles overall had significantly lower three-year OS and PFS compared with those with normal FLC levels; upon subclassification, only monoclonal sFLC abnormalities were significantly associated with reduced 3-year OS (62% vs 87%, p=0.008) and PFS (62% vs 84%, p=0.024), whereas polyclonal sFLC abnormalities showed no statistical significance (p=0.076 for OS; p=0.115 for PFS). These findings clarify the prognostic stratification value of sFLC abnormal subtypes, reinforce the prognostic utility of FLC profiling in DLBCL, and provide a more accurate criterion for clinically screening high-risk DLBCL patients, particularly those with monoclonal sFLC abnormalities who may be at higher risk for disease progression and mortality.

Studies by Maurer et al. (12) and Kim et al. (13) mentioned the association between FLC and DLBCL prognosis, but they did not combine molecular classification for in-depth analysis. In contrast, this study found that the adverse prognostic impact of monoclonal sFLC abnormalities was mainly concentrated in the non-GCB subtype—within this subtype, patients with monoclonal FLC abnormalities exhibited significantly poorer three-year OS compared with those with normal FLC profiles (3-year OS: 50% vs 83%, p=0.008)—while no statistically significant differences in three-year OS or PFS were observed in the GCB subtype between patients with normal versus abnormal FLC profiles (p=0.391 for OS; p=0.407 for PFS). This result reveals for the first time that the prognostic value of FLC abnormalities is “molecular subtype-dependent,” further supporting the association between monoclonal FLC and unfavorable survival outcomes; the lack of statistical significance in the GCB subtype may be attributable to the limited number of patients with abnormal FLC levels, particularly those with monoclonal abnormalities, so additional studies with larger GCB subtype cohorts are warranted to clarify the prognostic relevance of FLC in this population, and this finding also provides a basis for individualized risk assessment of different DLBCL subtypes. In addition the failure to confirm sFLC as an independent prognostic factor in multivariate analysis may be attributed to limited statistical power, requiring further validation with larger cohorts.

Activated B-Cell-like (ABC) DLBCL develops from post-GCB cells and is associated with the up regulation of Blimp-1, a transcription factor critical for plasma cell differentiation (15). Therefore, paraprotein production is more likely to occur during the development of the ABC-like subtype. Previous studies have shown that individuals with monoclonal FLC abnormalities are more likely to present ABC-like DLBCL, which accounts for about 73% of individuals with monoclonal FLC elevation and only 33% of individuals with normal FLC levels (14).

In this cohort, the non-GCB subtype accounted for 73.1% of patients with abnormal FLC levels and 57.8% of patients with normal FLC levels, consistent with previously reported distributions. Among patients diagnosed with DLBCL of both non-GCB and GCB subtypes, abnormal FLC levels were associated with an advanced disease stage, high intermediate to high IPI scores, and bone marrow infiltration. In the non-GCB subgroup, polyclonal FLC abnormalities were associated with advanced disease stage only, while monoclonal FLC abnormalities were additionally associated with intermediate to high IPI scores, bone marrow infiltration, and elevated LDH levels. These findings indicate that monoclonal FLC abnormalities in non-GCB DLBCL reflect a high tumor burden.

This dual-center study has several limitations. A major limitation of our study is the use of the Hans criteria (immunohistochemistry) instead of gene expression profiling to define GCB/non-GCB subtypes. While the non-GCB subtype largely overlaps with the ABC subtype, we cannot rule out heterogeneity within the non-GCB cohort or fully confirm the association between sFLC secretion and the ‘true’ ABC genotype. Future prospective studies using GEP for precise genotyping are needed to validate the relationship between ABC subtype and sFLC abnormalities. The retrospective design may introduce selection and information biases and the relatively short duration of follow-up may limit the robustness of long-term survival estimates. Moreover, ‘monoclonal FLC abnormality’ was determined based on the serum κ/λ FLC ratio. The existence of monoclonal components was not further verified by the immunofixation method (IFE), which has certain methodological limitations. Theoretically, in some cases, non-monoclonal factors (such as detection interference, transient B-cell activation) may cause slight abnormalities in the FLC ratio. However, in combination with the results in this study that ‘patients with monoclonal FLC all have characteristics of high tumor burden (such as 95.8% being III/IV stage and 79.2% with elevated LDH)’, the interference from non-monoclonal factors can be largely excluded. Future prospective studies can include IFE as a mandatory verification item for patients with abnormal FLC ratios, further improving the accuracy of monoclonal FLC diagnosis and providing more rigorous evidence support for its prognostic value.

## Conclusion

Abnormal serum FLC levels were associated with adverse clinical features and inferior survival outcomes in patients with DLBCL. Monoclonal FLC abnormalities, in particular, were indicative of high tumor burden and poor prognosis, especially within the non-GCB subtype. These findings support the use of serum FLC measurement as a convenient, non-invasive biomarker for prognostic assessment and disease monitoring in DLBCL. Additionally, serum FLC assessment may be valuable in detecting disease relapse. Further research is warranted to elucidate the mechanisms underlying FLC secretion in DLBCL, which may contribute to the development of novel DLBCL treatment strategies.

## Data Availability

The raw data supporting the conclusions of this article will be made available by the authors, without undue reservation.
